# Buruli Ulcer Surveillance, Benin, 2003–2005

**DOI:** 10.3201/eid1309.061338

**Published:** 2007-09

**Authors:** Ghislain Emmanuel Sopoh, Roch Christian Johnson, Annick Chauty, Ange Dodji Dossou, Julia Aguiar, Olivier Salmon, Françoise Portaels, Kingsley Asiedu

**Affiliations:** * Centre de Dépistage et de Traitement de l’Ulcère de Buruli, d’Allada, Bénin; † Programme National de Lutte contre l’Ulcère de Buruli, Cotonou, Bénin; ‡ Centre de Dépistage et de Traitement de l’Ulcère de Buruli de Pobè, Pobè, Bénin; § Centre de Dépistage et de Traitement de l’Ulcère de Buruli de Lalo, Lalo Bénin; ¶ Centre Sanitaire et Nutritionnel Gbemontin, Zagnanado, Bénin; # Hôpital “La Croix,” de Zinvié, Bénin; ** Institut de Médecine Tropicale, Antwerp, Belgium; †† World Health Organization, Geneva, Switzerland

**Keywords:** Buruli ulcer, surveillance, mapping, BU02, Benin, dispatch

## Abstract

We reviewed Buruli ulcer (BU) surveillance in Benin, using the World Health Organization BU02 form. We report results of reliable routine data collected on 2,598 new and recurrent cases from 2003 through 2005.

Buruli ulcer (BU), a disease caused by *Mycobacterium ulcerans*, is one of the recently classified 13 neglected tropical diseases (*1*). It has been reported in >30 countries (*2*). The disease starts as a nonulcerative lesion-like nodule, plaque, or edema. Without treatment, these early lesions will progress to an ulcer. The disease affects the bone in an estimated 13% of patients (*3*). Treatment often requires multiple interventions, including specific antimicrobial agents, surgery, and physiotherapy. Recurrence is high in many countries (*4*,*5*), and the economic effects on affected households, communities, and the health system are considerable (*6*,*7*). The exact mode of transmission of the causative organism is not known. The only known risk factors are related to water, particularly the use of unsafe water (*8*), especially that obtained from swamps (*9*).

We describe Benin’s surveillance system for BU from 2003 through 2005. The system is based on the use of the World Health Organization (WHO) BU02 form.

## The Study

The study took place in Benin, West Africa. The BU control activities are organized by a National Control Programme. Five BU Detection and Treatment Centers (CDTUB) are distributed throughout the BU-endemic regions. The detection, referral, and follow-up of BU cases rely heavily on community-based surveillance teams composed of village volunteers and 1 or 2 teachers and supervised by health workers from the nearest health facility.

The BU02 form acts as a triple registry. A trained nurse registers each case on the form. Each quarter, the completed first sheet is sent to the national level. The second sheet is sent to the regional level, and the third is kept at the CDTUB for local analysis. A training workshop is performed annually for the surveillance team. At the national level, data are computerized for analysis and mapping, and feedback is provided annually at a review meeting with all BU management participants.

With the use of this system, from January 1, 2003, through December 31, 2005, a total of 2,598 new and recurrent cases were reported and treated in Benin (Tables 1 and 2). The rates of disease recurrence (6%) were much lower than the figures reported in other countries, e.g., 16% in Ghana (*4**,**5*). Euverte found a rate of 3% recurrence among 103 patients treated with streptomycin and rifampin in Oueme, Benin, in 2005 (*6*). During the same period, the total numbers of leprosy and tuberculosis cases were 1,163 and 8,556, respectively. Thus, BU has become the second most important mycobacterial disease after tuberculosis in some endemic countries, including Benin (*3*) and Ghana (*7*).

**Table 1:** Monthly trends for Buruli ulcer cases, Benin, 2003–2005

Year	Jan	Feb	Mar	Apr	May	Jun	Jul	Aug	Sep	Oct	Nov	Dec	Total (DR/10,000 inhabitants)*
2003	59	62	48	49	52	63	79	44	41	93	64	77	731 (1.56)
2004	86	60	87	57	75	73	77	56	44	77	60	70	822 (1.73)
2005	72	89	89	91	93	100	77	94	73	88	97	82	1,045 (2.13)
Total	217	211	224	197	220	236	233	194	158	258	221	229	2,598

*DR, detection rate.

**Table 2:** Buruli ulcer cases reported in Benin by region, 2003–2005*

Region	2003, no. (%)	2004, no. (%)	2005, no. (%)	Total, no. (%)
Atlantique	171 (23)	171 (21)	263 (25)	605 (23)
Collines	2 (0)	0	0	2 (0)
Couffo	89 (12)	107 (13)	128 (12)	324 (12)
Littoral	8 (1)	18 (2)	31 (3)	57 (2)
Mono	14 (2)	13 (2)	20 (2)	47 (2)
Oueme	275 (38)	252 (31)	304 (29)	831 (32)
Plateau	26 (4)	43 (5)	79 (8)	148 (6)
Zou	124 (17)	201 (24)	198 (19)	523 (20)
Nigeria	4 (1)	3 (0)	6 (1)	13 (1)
Togo	2 (0)	2 (0)	1 (0)	5 (0)
Not specified	16 (2)	12 (1)	15 (1)	43 (2)
Total	731 (100)	822 (100)	1,045 (100)	2,598 (100)

*Benin surveillance captures data from the neighboring countries of Nigeria and Togo.

Consistent with other studies (*10*), our study found that 51% of the 2,598 cases were in children <15 years of age. Cases were equally distributed between male (49.7%) and female (50.3%) patients.

Of the total case-patients, 1,644 (63.3%) reported lesions on their lower limbs; 524 (20.2%), lesions on their upper limbs; 231 (8.9%), lesions on their head, neck, or trunk; 19 (0.7%), lesions in the perineal region; and 160 (6.2%), lesions in multiple areas. The location of a lesion was not noted on the BU02 form for 20 (0.8%) case-patients.

Many researchers believe that because legs and arms are the most exposed parts of the body they are more likely to be injured or to be bitten by an insect that may be associated with transmission of *M. tuberculosis*. However, why some lesions occur in the perineum, which is the least exposed area, remains unclear. In some villages, persons take baths in the swamps while carrying out domestic activities such as washing clothes or dishes. Lesions around the head, neck, and trunk were present in 9% of patients and in the perineum in almost 1%. Although these percentages are small, managing the technical and cosmetic aspects of lesions in the head, neck (*11*), and perineal regions (*12*) is difficult in Benin, where plastic surgeons are not available.

Nonulcerative early lesions (nodule, edema, and plaques) occurred in 27% of the total cases. Ulcers and mixed forms (an ulcer and some other form of the disease) occurred in 72% of the cases, and single ulcerative lesions occurred in 54%. The clinical form was not properly recorded for 2% of cases. Our figure of 72% is lower than the 94% rate reported elsewhere for Benin from 1989 through 1996 (*13*). The real challenge in Benin is how to further reduce the percentage of ulcers and sustain such surveillance efforts.

Regarding infection involving bone, Debacker et al. (*3*) reported a rate of 13% among 1,700 patients treated at CDTUB Zagnanado from 1997 through 2001. However, our results showed that bone involvement occurred in 6% of cases. Bone infection is a consequence of late disease (*14*). As progress is made in reducing late disease, bone lesions should be reduced.

Laboratory confirmation of BU is not frequently performed before treatment is begun. Although WHO strongly recommends laboratory confirmation of cases, in practice not all cases require it. Our study shows that 50% of cases are confirmed by at least 1 laboratory method under routine conditions.

The geographic distribution of cases shows that the BU-endemic areas are confined to the southern half of the country, Most BU-endemic villages occur along the Oueme and Couffo Rivers (Appendix Figure 1, and Appendix Figure 2).

The Mono Region has the lowest incidence of BU in southern Benin. By contrast, the other BU-endemic regions are around rivers. This observation cannot be due to insufficiency of reporting because there is a CDTUB in the area and surveillance is good (Appendix Figure 1). Unlike previous reports from Benin, our results suggest that the Oueme Region is now the most endemic for BU, not the Zou Region (Appendix Figure 1). We believe that this finding may be due to the active community-level detection and antimicrobial drug treatment conducted by the new BU center established in the Ouémé/Plateau region in April 2004.

## Conclusions

The data provided by Benin’s BU surveillance system that used the BU02 form enabled the BU Program in Benin to reliably describe the epidemiologic situation, evaluate the results of actions, measure the results of the centers, and plan future interventions. The collected data are ≈98% complete. We conclude that the BU surveillance system is useful to the BU Program in Benin. Because the BU02 form has 3 parts, data can be submitted from the field without the difficulties of photocopying the pages of the register or entering the data in a computer, which may be problematic at a rural facility level. However, training and supervision of health workers are required.

## Supplementary Material

Appendix Figure 1 Distribution of Buruli ulcer cases at regional and village levels, Benin.

Appendix Figure 2 Concentration of Buruli ulcer cases along the major Benin rivers, the Oueme and Couffo.

## References

[1] Molyneux DH, Hotez PJ, Fenwick A. “Rapid-impact interventions”: how a policy of integrated control for Africa’s neglected tropical diseases could benefit the poor. PLoS Med. 2005;2:e336. Epub 2005 Oct 11. 10.1371/journal.pmed.0020336 16212468 PMC1253619

[2] World Health Organization. Buruli ulcer: *Mycobacterium ulcerans* infection. Geneva: the Organization; 2000.

[3] Debacker M, Aguiar J, Steunou C, Zinsou C, Meyers WM, Guedenon A, *Mycobacterium ulcerans* disease (Buruli ulcer) in rural hospital, Southern Benin, 1997–2001. Emerg Infect Dis. 2004;10:1391–8. 15496239 10.3201/eid1008.030886 PMC3320395

[4] Amofah G, Asamoah S, Afram-Gyening C. Effectiveness of excision of pre-ulcerative Buruli lesions in field situations in a rural district in Ghana. Trop Doct. 1998;28:81–3. 9594673 10.1177/004947559802800208

[5] Debacker M, Aguiar J, Steunou C, Zinsou C, Meyers WM, Portaels F. Buruli ulcer recurrence, Benin. Emerg Infect Dis. 2005;11:584–9. 15829198 10.3201/eid1104.041000 PMC3320346

[6] Euverte H. Interest of streptomycicin and rifampicin association (WHO recommendation) in the treatment of *Mycobacterium ulcerans* infection (Buruli ulcer): evaluation after one year among 103 patients in Benin [in French]. Thèse de Médecine, Faculté de Médecine, Université de Toulouse III; 2005: 1062.

[7] Amofah G, Bonsu F, Tetteh C, Okrah J, Asamoa K, Asiedu K, Buruli ulcer in Ghana: results of a national case search. Emerg Infect Dis. 2002;8:167–70. 10.3201/eid0802.010119 11897068 PMC2732443

[8] Johnson RC, Makoutodé M, Sopoh GE, Elsen P, Gbovi J, Pourteau LH, Buruli ulcer distribution in Benin. Emerg Infect Dis. 2005;11:500–1. 15789490 10.3201/eid1103.040597 PMC3298242

[9] Debacker M, Portaels F, Aguiar J, Steunou C, Zinsou C, Meyers W, Risk factors for Buruli ulcer, Benin. Emerg Infect Dis. 2006;12:1325–31. 17073079 10.3201/eid1209.050598 PMC3294727

[10] Raghunathan PL, Whitney EA, Tappero JW. Sam Bigri DAA, Amofah G, Asamoa K, et al. Burden of Buruli ulcer disease in Upper Denkyira District, Ghana, 1994–2000. Abstract: Report of the Meeting of the 4th Advisory Group Meeting on Buruli ulcer, 5–7 Mar 2001, Geneva, Switzerland. p. 72.

[11] Agbenorku P. BU in the head and neck region: reconstructive challenges. Abstract: Colloquium on improving access to TB and Buruli ulcer treatment in Africa. 5–7 Dec 2005, Cotonou, Benin.

[12] Sica A, Dekou A, Kaba L, Ouattara D, Kouame B, Konan PG, Genital sites of Buruli ulcer (BU): clinical and therapeutic aspects. Prog Urol. 2005;15:736–8. 16459698

[13] Aguiar J, Domingo MC, Guedenon A, Meyers WM, Steunou C, Portaels F. L’ulcère de Buruli, une maladie mycobactérienne importante et en recrudescence au Bénin. ARSOM Bulletin des Séances. 1997;3:325–56.

[14] Portaels F, Zinsou C, Aguiar J, Debacker M, de Biurrun E, Guedenon A, Les atteintes osseuses dans l’ulcère de Buruli: à propos de 73 cas. Bull Seances Acad R Sci Outre Mer. 2003;49:161–90.

