# Supplementary figures and images for: Buruli Ulcer Surveillance, Benin, 2003–2005
Source: Emerg Infect Dis. 2007 Sep;13(9):1374–6. doi: 10.3201/eid1309.061338 (PMC2857274; doi:10.3201/eid1309.061338)

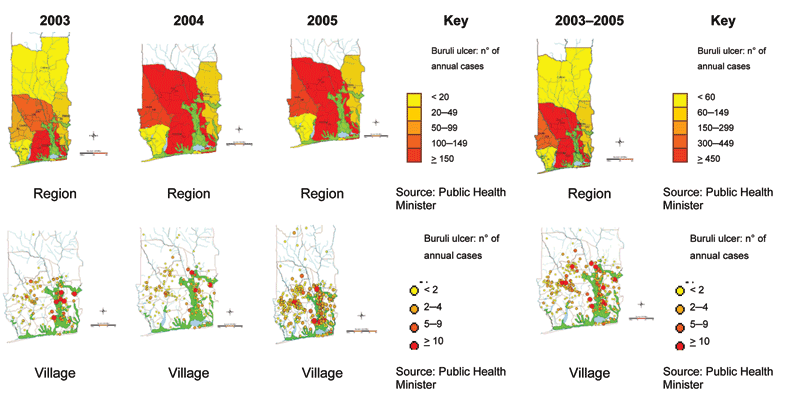

Supplement: Appendix Figure 1 — Distribution of Buruli ulcer cases at regional and village levels, Benin. [file 06-1338_appF1-s1.gif]

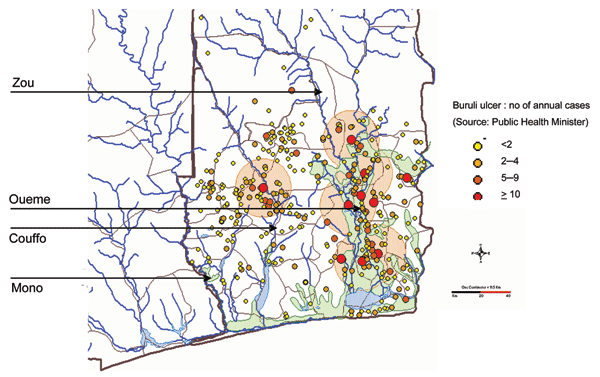

Supplement: Appendix Figure 2 — Concentration of Buruli ulcer cases along the major Benin rivers, the Oueme and Couffo. [file 06-1338_appF2-s2.gif]
